# Complement Component 4D (C4d) Staining in Lupus Nephritis: Correlating Renal Histopathological Features With Clinical Presentation and Short-Term Outcomes

**DOI:** 10.7759/cureus.112010

**Published:** 2026-07-03

**Authors:** Noureen Amin, Amit Bari, Muhammad Nazrul Islam, Mohammad Rezwanur Rahman, Gul Jannat, Mohammad Kamrul Hasan

**Affiliations:** 1 Nephrology, National Institute of Kidney Diseases and Urology, Dhaka, BGD; 2 Nephrology, Kidney Foundation Hospital, Dhaka, BGD; 3 Nephrology, Bangladesh Medical University (BMU), Dhaka, BGD; 4 Hepatology, National Gastroliver Institute and Hospital, Dhaka, BGD; 5 Nephrology, Holy Family Red Crescent Medical College, Dhaka, BGD

**Keywords:** c4d staining, clinical presentation, lupus nephritis, renal histopathology, short-term outcome, systemic lupus erythematosus disease activity index

## Abstract

Introduction

Systemic lupus erythematosus frequently manifests as lupus nephritis (LN), a major cause of renal morbidity. Standard markers lack specificity, making renal biopsy essential. Complement component 4D (C4d) staining, reflecting classical complement activation, correlates with histopathological lesions and outcomes. This study evaluates C4d's clinical relevance in biopsy-proven LN, emphasizing its potential in detecting disease severity.

Materials & methods

This observational study at Bangabandhu Sheikh Mujib Medical University (BSMMU), Dhaka, conducted from September 2021 to August 2022, enrolled 45 adults with biopsy-proven LN; 43 completed follow-up. Participants were selected based on revised American College of Rheumatology (ACR) criteria and underwent thorough clinical evaluation, serological testing, and ultrasound-guided renal biopsy. Histopathological assessment followed the International Society of Nephrology and the Renal Pathology Society (ISN/RPS) 2003 classification, including activity and chronicity indices and immunohistochemical C4d staining. Follow-up investigations were performed at two and six months.

Results

Among the 43 patients with LN (mean age 28.4 ± 7.7 years; 86% female subjects), 42 (97.7%) patients had hematuria and 25 (58.1%) had renal impairment. Class IV was the predominant histological subtype in 18 (41.9%) cases, with mild chronicity in 20 (71.4%) cases. C4d deposition, primarily arteriolar in 24 (55.8%) cases, correlated significantly with Systemic Lupus Erythematosus Disease Activity Index (SLEDAI) scores and histological activity indices (p<0.05). There is a significant relationship between C4d intensity and SLEDAI score, highlighting the prognostic utility of C4d staining.

Conclusion

The present study identifies C4d staining to be associated with active renal damage and higher SLEDAI scores, reflecting immune-mediated injury. These findings highlight its value as a potential marker that may enhance current histological assessment. Therefore, staining with C4d on renal biopsy may be emphasized for early detection of severity in patients with lupus.

## Introduction

Systemic lupus erythematosus (SLE) is a chronic autoimmune disease characterized by multisystem involvement and the production of autoantibodies [1]. Lupus nephritis (LN), a severe manifestation of SLE, affects up to 60% of patients during the disease course and significantly contributes to morbidity and mortality [2]. The diagnosis of LN is based on clinical features such as proteinuria, hematuria, and impaired renal function, supported by serological markers, including anti-dsDNA antibodies and hypocomplementemia [3]. However, these markers often lack sensitivity and specificity in reflecting renal pathology, necessitating histopathological evaluation through renal biopsy [4]. Renal biopsy remains the gold standard for diagnosing LN, enabling classification according to the International Society of Nephrology/Renal Pathology Society (ISN/RPS) system and guiding therapeutic decisions [5]. Histopathological features commonly observed in LN include mesangial hypercellularity, endocapillary proliferation, wire loop lesions, crescents, and varying degrees of interstitial fibrosis and tubular atrophy [6]. These features are critical for assessing disease activity and chronicity, which in turn influence prognosis and treatment strategies.

Complement activation via the classical pathway plays a central role in LN pathogenesis, and complement component 4D (C4d), a stable degradation product of complement component C4, serves as a footprint of classical pathway and/or lectin pathway activation [7]. Immunohistochemical staining for C4d has emerged as a potential tool for evaluating complement-mediated injury in renal tissues. Studies have demonstrated that the exact value of C4d based on its distinct compartmental localization (glomerular vs arteriolar vs peritubular) remains a subject of debate [8,9]. The prognostic value of C4d staining has also been explored in relation to short-term outcomes. A prospective study by Bheemavathi et al. indicated that C4d positivity was observed in 70% of LN cases, although the correlation with activity index was not statistically significant (p=0.4), highlighting the need for further investigation [10]. Nonetheless, the reproducibility and stability of C4d as a marker make it a promising adjunct to conventional histopathological assessment. This underscores the relevance of incorporating C4d staining into routine renal biopsy evaluation in resource-limited settings, where early prognostication can inform timely therapeutic interventions.

Given the limitations of conventional serological markers and the invasive nature of repeat biopsies, C4d staining offers a valuable, reproducible, and potential tool in the management of LN. This study aims to correlate C4d staining patterns with renal histopathological features and disease severity at baseline and the sixth month following treatment in patients with biopsy-proven LN. This study also focuses on the association between the intensity of C4d staining and outcome at the sixth month in patients with lupus.

## Materials and methods

Study design

This observational study was conducted in the Department of Nephrology, Bangabandhu Sheikh Mujib Medical University (BSMMU), Dhaka, Bangladesh, between September 01, 2021 and August 31, 2022.

Study participants

As per the selection criteria, 45 adult patients (age≥18 years) with diagnosed SLE and LN, according to the revised American College of Rheumatology (ACR) criteria [11], admitted to the department of Nephrology, BSMMU, were enrolled in the study through a purposive sampling technique. Data were collected through face-to-face interviews using a semi-structured questionnaire and data collection tools. Patients with malignancy, active infection, other causes of glomerulopathies (infections such as HIV, hepatitis B or C virus, malignancy, drugs), other autoimmune diseases, patients with inadequate kidney biopsy specimen (<5 scorable glomeruli), patients with baseline serum creatinine ≥3.0 mg/dl, patients unwilling to give consent, pregnant women, and lactating mothers were excluded from the study. The study population underwent detailed history-taking, physical examination, and relevant investigations. Participants were counseled on LN and provided consent before undergoing ultrasound-guided renal biopsy.

Baseline tests included complete blood count (CBC), urine routine and microscopy examination (urine R/M/E), urine protein to creatinine ratio (u-PCR), serum creatinine, albumin, anti-dsDNA, anti-phospholipid antibody, and C3/C4. Renal histology was classified according to the ISN/RPS 2003 LN classification. Disease activity was assessed using the Systemic Lupus Erythematosus Disease Activity Index (SLEDAI) and categorized accordingly [12]. Treatment followed Kidney Disease: Improving Global Outcomes (KDIGO) guidelines [12]. Follow-ups at two and six months repeated prior tests, with data documented and patient contact maintained to minimize drop-out.

Two renal tissue cores were obtained for light microscopy and direct immunofluorescence (DIF) microscopy, then analyzed at Armed Forces Institute of Pathology, Dhaka (AFIP) for histopathological study using ISN/RPS 2003 LN classification. The biopsy samples were examined by an experienced renal histopathologist with standard staining as well as C4d staining, who was blinded to clinical & outcome data. C4d was detected by immunohistochemistry on formalin-fixed, paraffin-embedded tissue using rabbit anti-human C4d polyclonal antibodies.

Histopathology included activity/chronicity indices (max 24/12) and the intensity of C4d was scored as mild (<10% of tissue specimen), moderate (10-50% of tissue specimen) and severe (>50% of tissue specimen). Out of the 45 respondents enrolled for the study, two were lost to follow up. Final data analysis was done for 43 patients. Outcome was categorized into complete remission, partial remission and no remission based on proteinuria and serum creatinine as defined by the KDIGO guideline. Complete response was defined by reduction in proteinuria <0.5 g/g (50 mg/mmol) measured as the PCR from a 24-hour urine collection, stabilization or improvement in kidney function (±10-15% of baseline), within six to 12 months of starting therapy, but could take more than 12 months. Partial response was defined by reduction in proteinuria by at least 50% and to <3 g/g (300 mg/mmol) measured as the protein/creatinine ratio (PCR) from a 24-hour urine collection, stabilization or improvement in kidney function (± 10-15% of baseline) within six to 12 months of starting therapy. No remission was defined by failure to achieve a partial or complete response within six to 12 months of starting therapy.

Ethical consideration

Before starting this study, the research protocol was submitted to the Institutional Review Board (IRB) of BSMMU, Dhaka and approval was obtained (no. 3778). A voluntary informed written consent was taken from every patient after an explanation of the procedure and purpose of the study. Every patient was given the right to participate or refuse to participate. Every patient had the right to withdraw from the study at any time without compromising their medical care. The patient's privacy was ensured and the patient's information was not disclosed to anyone. This study does not cause any harm to the patient and does not affect the quality of treatment. The patient and/or responsible family member was informed about the potential risks, and no drug or placebos were used.

Statistical analysis

Statistical analysis was performed using IBM SPSS Statistics for Windows, Version 25 (Released 2017; IBM Corp., Armonk, New York, United States). After collection, all the data were checked and cleaned. Quantitative data were expressed as percentage, mean and standard deviation and qualitative data were expressed as frequency distribution and percentage. To determine statistical significance, one-way ANOVA and Chi-square test were considered according to applicability. p value<0.05 was considered statistically significant.

## Results

The study population included 43 patients with a mean age of 28.4 ± 7.7 years, predominantly 37 (86.0%) female patients (Table 1).

**Table 1:** Descriptive statistics of the study population (n=43) Data presented as n (%) or mean ± SD; SLEDAI: Systemic Lupus Erythematosus Disease Activity Index; u-PCR: urine protein to creatinine ratio.

Characteristics		Data
Age (in years)		28.4 ± 7.7
Age group (in years)	≤20	9 (20.9%)
	21 – 30	19 (44.2%)
	31 – 40	13 (30.2%)
	41 – 50	2 (4.7%)
Gender	Male	6 (14.0%)
	Female	37 (86.0%)
Clinical presentation during admission	Hematuria	42 (97.7%)
	Renal impairment	25 (58.1%)
	SLEDAI	15.2 ± 3.2
Biochemical profile during admission	u-PCR (≥ 3.5 g/day)	25 (58.1%)
	Anti dsDNA	40 (93.0%)
	Low C3	42 (97.7%)
	Low C4	40 (93.0%)
	Positive anti-phospholipid antibody	11 (25.6%)

Hematuria was the most common clinical presentation in 42 (97.7%) cases, with renal impairment seen in 25 (58.1) cases. Disease activity was reflected by a mean SLEDAI score of 15.2 ± 3.2. Biochemical assessment showed u-PCR ≥3.5 g/day in 25 (58.1%) cases, while 40 (93.0%) cases had anti-dsDNA antibodies. Complement deficiencies were frequent, with low C3 in 42 (97.7%) cases and low C4 in 40 (93.0%) cases. Additionally, 11 (25.6%) patients tested positive for anti-phospholipid antibodies.

The renal histopathological evaluation revealed diverse LN classifications, with Class IV being the most common 18 (41.9%) cases, followed by Class III 10 (23.3%) cases (Table 2).

**Table 2:** Renal histopathology of the study population at baseline (n=43) Data presented as n (%) or mean ± SD.

Characteristics		Data
Lupus nephritis (LN)	Class I	3 (7.0%)
	Class II	5 (11.6%)
	Class III	10 (23.3%)
	Class IV	18 (41.9%)
	Class V	4 (9.3%)
	Class VI	3 (7.0%)
Activity indices	Mild (0-8)	8 (28.6%)
	Moderate (9-16)	13 (46.4%)
	Severe (17-24)	7 (25.0%)
Chronicity indices	Mild (0-4)	20 (71.4%)
	Moderate (5-8)	5 (17.9%)
	Severe (9-12)	3 (10.7%)
Tubulointerstitial involvement	≤10%	10 (23.3%)
	11-25%	12 (27.9%)
	26-50%	16 (37.2%)
	˃50%	5 (11.6%)
C4d deposition site	Arteriolar	24 (55.8%)
	Tubular basement membrane	2 (4.7%)
	Glomerular	10 (23.3%)
	Peritubular capillary	7 (16.3%)
C4d deposition intensity	Mild	7 (16.3%)
	Moderate	15 (34.9%)
	Severe	21 (48.8%)
Outcome	Complete remission	14 (32.6%)
	Partial remission	15 (34.9%)
	No remission	14 (32.6%)

Moderate disease activity was observed in 13 (46.4%) of cases. Chronicity indices indicated that 20 (71.4%) patients had mild chronic changes, with moderate and severe forms present in five (17.9%) and three (10.7%) cases. Tubulointerstitial involvement varied, with 16 (37.2%) cases having 26-50% damage. C4d deposition was common in arteriolar sites 24 (55.8%) cases, with glomerular involvement in 10 (23.3%) cases. Severe C4d deposition occurred in 21 (48.8%) of cases. Clinical outcomes showed 15 (34.9%) cases achieved partial remission, 14 (32.6%) cases complete remission, while 14 (32.6%) cases had no remission, highlighting variability in disease progression.

**Figure 1:**
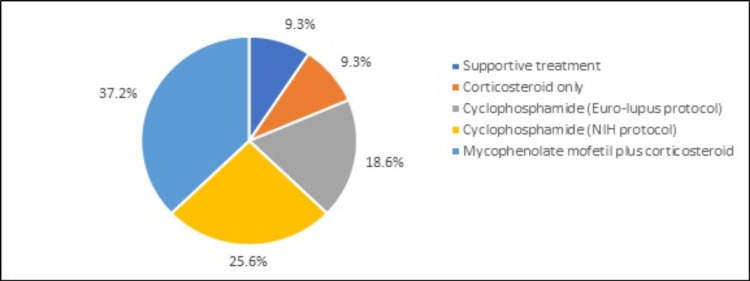
Distribution of study population according to treatment received following renal biopsy

Among 43 patients, 16 (37.2%) cases received supportive therapy (Figure 1), while immunosuppressive regimens varied.

The National Institutes of Health (NIH) protocol [12] was used in 11 (25.6%) cases, the Euro-lupus protocol [12] in eight (18.6%) cases, and mycophenolate mofetil with corticosteroids in 4 (9.3%) cases. Corticosteroids alone were administered in four (9.3%) cases, reflecting individualized LN management.

**Figure 2:**
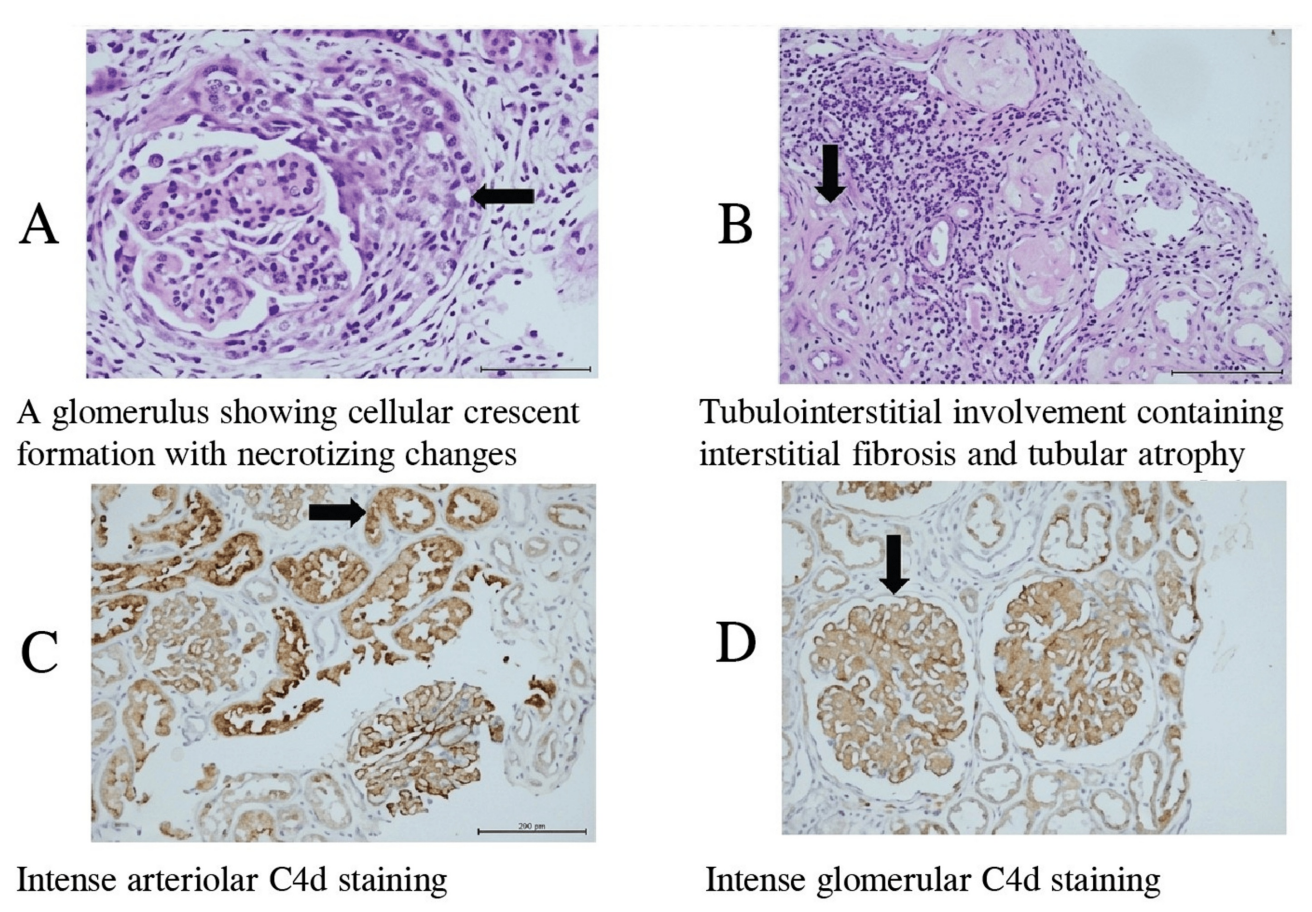
Renal biopsy samples obtained from the study population

Renal biopsy findings revealed that glomerular injury was evident through crescent formation and necrotizing changes (Figure 2A), while tubulointerstitial damage is marked by fibrosis and atrophy (Figure 2B).

Immune deposition patterns showed intense C4d staining in arteriolar and glomerular sites, highlighting complement activation (Figure 2C). Crescent formation signaled active inflammation, while tubulointerstitial fibrosis indicated chronic progression (Figure 2D).

**Figure 3:**
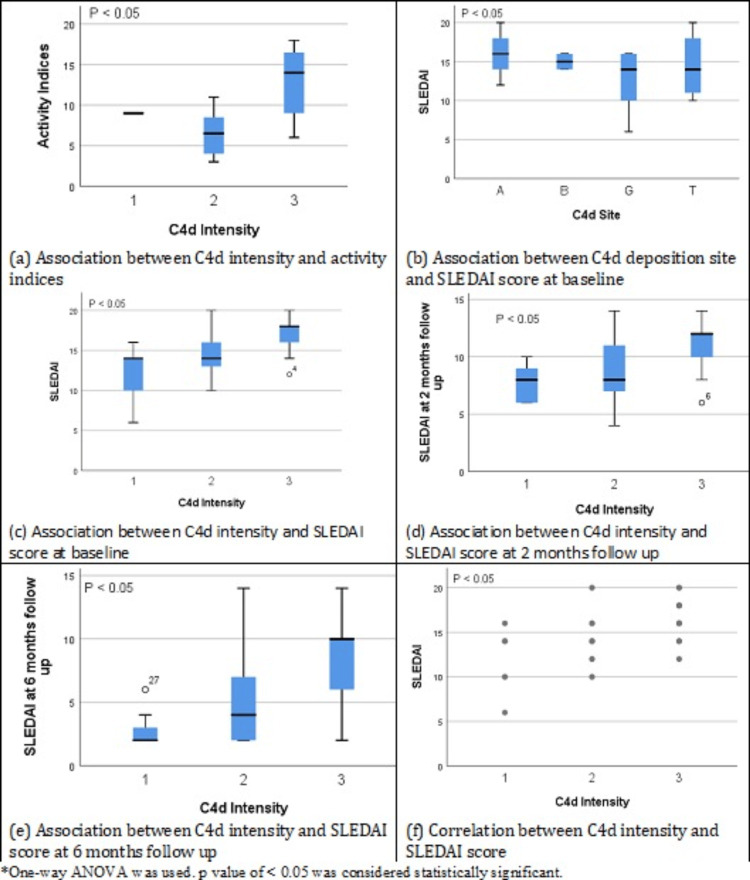
Relation of C4d staining with renal histopathological features SLEDAI: Systemic Lupus Erythematosus Disease Activity Index.

The relationship between C4d staining intensity and key clinical and histopathological features in LN was assessed in 43 patients (Figure 3).

The analysis revealed significant associations between C4d deposition and disease activity markers. Figure 3a demonstrated that higher C4d intensity correlated with increased activity indices (p<0.05), indicating its potential role in disease severity. Figure 3b highlighted variations in SLEDAI scores based on C4d deposition sites, with notable differences among affected regions (p<0.05). Figure 3c showed a direct correlation between C4d intensity and baseline SLEDAI scores (p<0.05), supporting its relevance in initial disease assessment. Figure 3d illustrated that higher C4d intensity remains associated with elevated disease activity at two months follow-up (p<0.05), while Figure 3e extended this trend to six months (p<0.05), emphasizing its role in monitoring disease progression. Figure 3f provided a scatter plot showing a significant positive correlation between C4d intensity and SLEDAI scores over time (p<0.05), suggesting sustained complement activation as a contributor to persistent inflammation.

**Figure 4:**
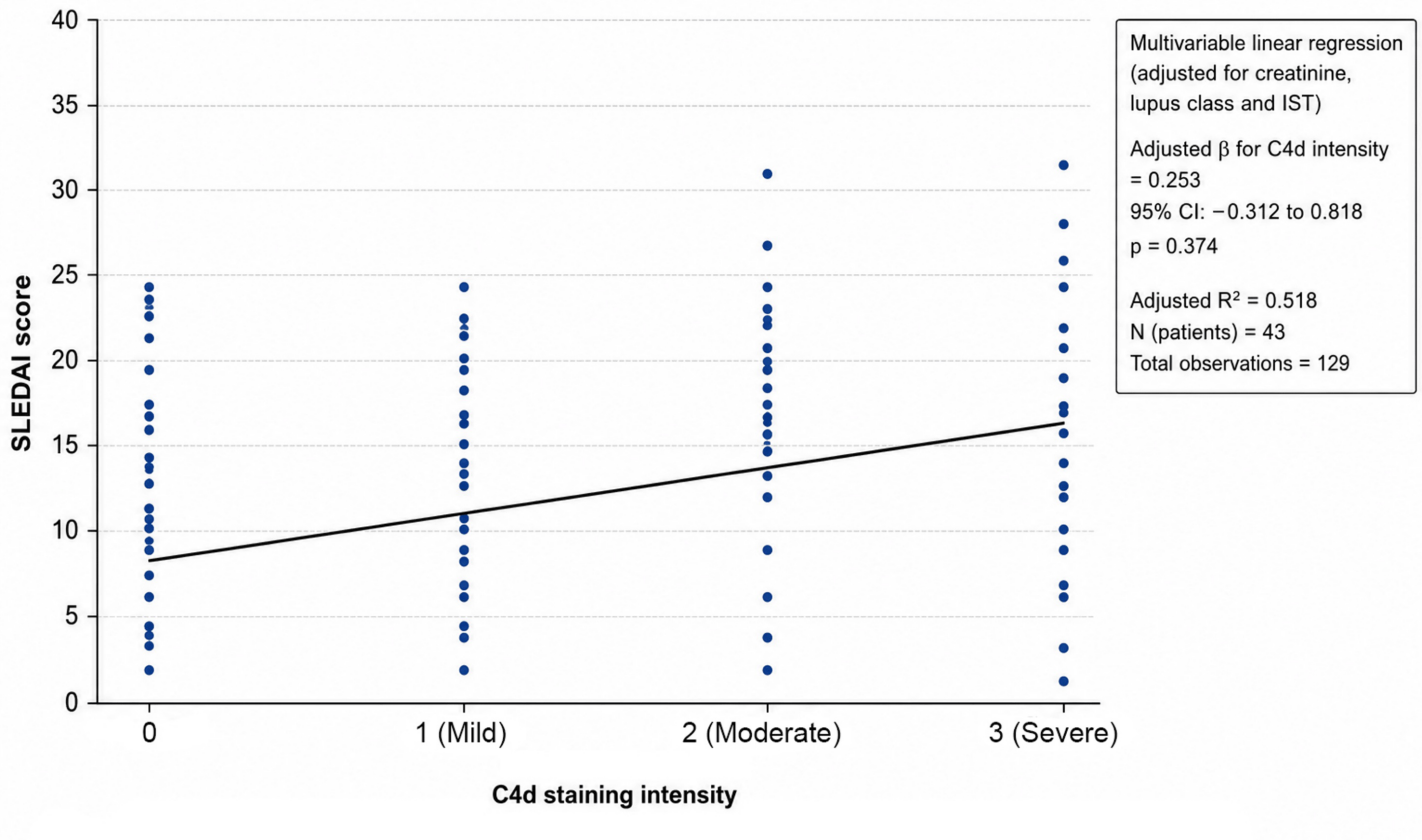
Relation of C4d intensity with SLEDAI scores at baseline & follow-ups SLEDAI: Systemic Lupus Erythematosus Disease Activity Index.

Scatter plots demonstrated the relationship between C4d intensity and observed SLEDAI scores, while the fitted regression lines represented the adjusted predicted values from the multivariable models (Figure 4).

Although positive trends were observed, C4d intensity was not independently associated with disease activity after adjustment for the confounding variables (serum creatinine, LN class, and immunosuppressive therapy).

The relationship between C4d deposition and clinical outcomes at six months was analyzed (Table 3).

**Table 3:** Distribution of study population according to relation between C4d staining and outcome (n=43) Data presented as n (%) ^a^Chi-squared test was used. p value <0.05 was considered statistically significant.

Characteristics		Outcome at the sixth month			p
		Complete remission	Partial remission	No remission	
C4d deposition site	Arteriolar	4 (16.7%)	10 (41.7%)	10 (41.7%)	0.283
	Glomerular	5 (50.0%)	3 (30.0%)	2 (20.0%)	
	Peritubular capillary	4 (57.1%)	1 (14.3%)	2 (28.6%)	
	Tubular basement membrane	1 (50.0%)	1 (50.0%)	0	
C4d deposition intensity	Mild	5 (71.4%)	1 (14.3%)	1 (14.3%)	<0.05^a^
	Moderate	7 (46.7%)	6 (40.0%)	2 (13.3%)	
	Severe	2 (9.5%)	8 (38.1%)	11 (52.4%)	

Arteriolar deposition was associated with no remission in 10 (41.7%) of cases, while glomerular and peritubular capillary depositions showed higher rates of complete remission in five (50.0%) and four (57.1%) cases respectively. Tubular basement membrane deposition was rare, with equal distribution between complete and partial remission. C4d intensity significantly influenced prognosis (p<0.05), with mild deposition associated with complete remission in five (71.4%) cases, whereas severe deposition was linked to no remission in 11 (52.4%) cases.

## Discussion

The study population exhibited a mean SLEDAI score of 15.2 ± 3.2, indicating high disease activity. This aligns with findings by Koelmeyer R. et al., who reported that patients with SLEDAI scores ≥10 experienced more severe disease manifestations [13]. However, the present study population demonstrated a higher prevalence of anti-dsDNA positivity (93.0%), compared to the 44% reported in a study on circulating S100 proteins in SLE patients [14], suggesting a stronger autoimmune response. In the present study, 97.7% of patients with LN presented with hematuria, a prevalence notably higher than the 85% reported by Yates et al. in a multicenter study on LN cohorts [15]. This suggests a more severe renal involvement in our population. Additionally, renal impairment was observed in 58.1% of cases, aligning closely with findings from Mok et al., who reported renal dysfunction in 55% of patients with LN [16]. Complement deficiencies were frequent, with low C3 in 97.7% and low C4 in 93.0%, consistent with previous reports linking low C4 to distinct clinical implications in SLE [17].

The predominance of Class IV LN (41.9%) is consistent with findings by Wakasugi et al., who reported a high prevalence of Class III and IV nephritis in SLE patients [18]. Bolognesi et al. reported similar distributions, with Class IV being the most frequent [19]. Moderate disease activity was observed in 46.4% of cases, which is comparable to the trends noted by Moroni et al., who highlighted the predictive value of chronicity indices in LN progression [20]. The chronicity indices in our cohort revealed mild chronic changes in 71.4% of patients, with moderate and severe forms present in 17.9% and 10.7%, respectively. These results align with prior studies showing the correlation between chronicity indices and long-term renal outcomes [21]. The tubulointerstitial involvement observed in this study, with 37.2% of cases showing 26-50% damage, is lower than findings from prior studies showing moderate to severe tubulointerstitial inflammation in 69.3%-72% of LN biopsies [22], suggesting potential differences in patient cohorts or biopsy criteria. C4d deposition was common in arteriolar sites (55.8%), with glomerular involvement in 23.3%, similar to findings from Jiang et al., who reported C4d deposition as a marker of disease progression in IgA nephropathy [22]. Severe C4d deposition (48.8%) further supports its role in complement activation and renal injury [23]. Varied clinical outcomes reflect the heterogeneity in LN progression.

The renal biopsy findings in this study demonstrate significant glomerular injury characterized by crescent formation and necrotizing changes, consistent with previous reports on cellular crescents as markers of severe glomerular injury, correlating with disease progression [24-26]. Tubulointerstitial fibrosis and atrophy, indicative of chronic kidney disease progression, align with findings from LN studies [27]. The immune deposition patterns observed, particularly intense C4d staining in arteriolar and glomerular sites, highlight complement activation, consistent with prior studies suggesting C4d staining as a reliable marker of immune-complex deposits in LN [9].

The present study supports earlier findings on the clinical relevance of C4d staining in LN. Ding et al. [9] observed C4d deposition in 98.8% of renal biopsies, especially in glomeruli, tubular basement membranes, and arterioles. Their data also linked tubular basement membrane staining to higher disease activity, aligning with our observation of increased activity indices at higher C4d intensity (p<0.05). Zickert et al. found that plasma C4d levels correlate with renal activity and treatment response in proliferative LN [28], supporting our observation of SLEDAI score variation with tissue C4d deposition. In contrast, Malakoutian et al. proposed that C4d serves more as a prognostic marker than a reflection of current disease activity [29], differing from our findings linking C4d intensity to disease severity.

The findings of the present study demonstrate a sustained correlation between C4d staining intensity and SLEDAI scores at baseline and during follow-up, suggesting that C4d may serve as a potential marker of disease activity in LN. This aligns with the study by Ding et al., which reported that tubular basement membrane C4d deposition was significantly associated with higher SLEDAI scores [9].

Our findings highlight that the intensity of C4d deposition are associated with clinical outcomes in LN. Arteriolar deposition was observed in poorer remission, consistent with Ding et al., who linked such patterns to adverse vascular pathology [9]. In contrast, glomerular and peritubular C4d deposition were linked to higher complete remission in our study, echoing Zickert et al.'s findings of improved renal scores with decreased C4d levels post-treatment [27]. C4d intensity also showed prognostic value: mild deposition predicted favorable outcomes, supporting Kraaij et al., who found that declining plasma C4d level reflected therapeutic response [30].

However, the observed association between C4d staining intensity and SLEDAI is largely attributable to underlying disease severity and treatment-related factors rather than an independent effect of C4d deposition. As enhanced complement activation promotes glomerular damage, contributing to disease progression, so increased C4d deposition likely reflects the severity of the underlying renal pathology rather than acting as an independent indicator of clinical disease activity. Patients with more advanced histopathological classes of LN generally exhibit greater complement activation, higher serum creatinine levels, and require more intensive immunosuppressive therapy. The close relationship among these factors may explain why C4d staining intensity lost statistical significance after adjustment for these established markers of disease severity.

The study has several key limitations that should be considered when interpreting the results. These include small sample size, a single-center design, the heterogeneity of treatment or lack of adjustment in treatment, small subgroup size for C4d deposition site, lack of standardization of C4d scoring and the use of purposive (non-random) sampling, which may introduce bias. Additionally, the follow-up period was relatively short, limited to just six months, and there was no external validation cohort to support the findings. To validate the clinical relevance of C4d staining, larger, multicenter studies incorporating multi-marker immune panels and longer follow-up periods are needed.

## Conclusions

This study demonstrated a high burden of disease activity among patients with LN, with a predominance of Class IV disease and frequent complement abnormalities. Increased C4d staining intensity was significantly associated with active lesions in renal histopathology. Higher C4d deposition also correlated with elevated SLEDAI scores and greater complement consumption, reflecting ongoing immune-mediated renal injury. These findings support that C4d deposition reflects the severity of intrarenal complement activation and inflammatory activity. Therefore, C4d staining may serve as a valuable adjunctive histopathological marker for assessing disease activity in LN alongside conventional clinical and pathological prognostic parameters.
